# The COVID-19 pandemic and OBGYN residency training: We have a problem and it’s not just masks

**DOI:** 10.1186/s12909-024-05364-8

**Published:** 2024-04-05

**Authors:** Alexandria C. Kraus, Anthony Bui, Kimberly Malloy, Jessica Morse, Omar M. Young

**Affiliations:** 1 Division of Maternal Fetal Medicine, Department of Obstetrics and Gynecology, University of North Carolina at Chapel Hill, 3010 Old Clinic Building, Chapel Hill, NC 27599 USA; 2 Department of Obstetrics and Gynecology, University of North Carolina at Chapel Hill, Chapel Hill, USA; 3 Division of Family Planning, Department of Obstetrics and Gynecology, University of North Carolina at Chapel Hill, Chapel Hill, USA

**Keywords:** Residency, Obstetrics and gynecology, COVID-19, Surgical training, Mental health, Wellness, United States

## Abstract

**Background:**

The COVID-19 pandemic has left no one untouched. Resident trainees have been driven to reconsider virtually every component of their daily lives. The purpose of this pilot study is to evaluate the impact of the COVID-19 pandemic on Obstetrics and Gynecology (OBGYN) residency training and education.

**Methods:**

A cross-sectional pilot study was conducted between 2/2022 and 5/2022. A survey was created and distributed to OBGYN residents. The survey queried the effects of the pandemic on OBGYN residents’ procedure skills training and mental health.

**Results:**

A total of 95 OBGYN residents across programs affiliated with each American College of Obstetricians and Gynecologists (ACOG) district participated in the survey. Among them, just over half (*n* = 52, 55%) self-identified as under-represented minorities. A significant majority, 80% (*n* = 81), felt their gynecological training was inadequate, with 70% of fourth-year residents expressing a lack of confidence in their ability to independently practice gynecology after graduation. This lack of confidence among fourth-year residents suggests a notable disparity in readiness for independent gynecological practice, linked to meeting ACGME requirements before completing their residency (*p* = 0.013). Among the residents who reported a negative impact of the pandemic on their mental health (*n* = 76, 80%), about 40% (*n* = 31) had contemplated self-harm or knew a colleague who considered or attempted suicide (*p* < 0.001). This issue was especially pronounced in residents experiencing burnout (*n* = 44, 46%), as nearly half (*n* = 19, 43%) reported suicidal thoughts or knew someone in their program who had such thoughts or engaged in self-harm (*p* = 0.048).

**Conclusions:**

Residents expressed concerns about reduced hands-on gynecological training and doubts about their readiness for independent practice post-residency, highlighting the need for enhanced support through mentorship and revised training curriculums. Additionally, despite the availability of mental health resources to address pandemic-induced burnout, their underuse suggests a need for more accessible time for residents to use at their discretion and flexible training schedules that encourage mental health support resource utilization.

**Supplementary Information:**

The online version contains supplementary material available at 10.1186/s12909-024-05364-8.

## Introduction

The disease known as severe acute respiratory syndrome coronavirus 2 (SARS-CoV-2) was first described in China in December of 2019 [1], and in March of 2020, the World Health Organization (WHO) declared the SARS-CoV-2 (i.e., COVID-19) outbreak a pandemic [2]. The disease has left no one untouched. The healthcare industry specifically has been overwhelmed by the effect of COVID-19 on resources with providers driven to reconsider virtually every component of their daily lives and practice. To sustain adequate hospital resources, elective surgical procedures were cancelled, and clinical volumes were dramatically reduced. Telemedicine was utilized to provide a significant portion of outpatient healthcare and inpatient care teams were condensed.

Resident schedules, in particular, were modified to provide a workforce where necessary and educational curricula transitioned toward virtual platforms in attempts to avoid exposures and to enforce social distancing [3]. While virtual solutions were implemented to counteract missed in-person pedagogic didactics and conferences, there were no immediate substitutes for the significant reduction of hands-on clinical and surgical experiences during this period. Additionally, visitor restrictions impacted the development of resident communication skills and emotional intelligence. Infected residents often required long absences, which resulted in re-assignments of remaining residents and trickle-down effects on overall residency training and education. Nonetheless, limited studies have been published on the impact of COVID-19 on residency training [4–6], and specifically, in the field obstetrics and gynecology (OBGYN) training [7–10]. Therefore, the purpose of this pilot study is to examine the impact of the COVID-19 pandemic on OBGYN residency training and education.

## Methods

A nationwide, cross-sectional pilot study was conducted between February 2022 and May 2022. An anonymous survey was created using Qualtrics XM, (an online, secure survey platform), and OBGYN residents across the United States were invited to participate. The survey was preceded by a statement (1) explaining the purpose of the survey, (2) clarifying that the data would be de-identified before analysis and (3) delineating that program leadership would not have access to the responses. There were no incentives to participate. The study was reviewed and determined to be exempt by the Institutional Review Board (IRB #22–0136). A link to the survey was e-mailed to OBGYN program directors and program managers with a request that it be forwarded to all the residents in their program. The emails of the program directors and program managers were obtained from the Association of Professors of Gynecology and Obstetrics (APGO) website [11]. Reminder emails to encourage participation were distributed every four weeks for a period of three months. Recruitment posts were placed on social media as well. Responses were captured anonymously to maintain confidentiality.

All OBGYN residents in the United States were eligible to participate [12]; however, it is unclear how many residents received access to the survey, as there was limited verification from residency program leadership confirming distribution. In addition, multiple emails to both program directors and program managers were returned as invalid, further suggesting that many residency programs did not receive access to the survey at all. As such, it is difficult to report an accurate response rate.

The 28-question survey was developed after a comprehensive examination of the contemporary literature and following a review by local content experts to improve overall quality as well as to ensure content validity among assessed domains. Attention was paid to the Accreditation Council for Graduate Medical Education (ACGME) core competencies [13], and in particular, patient care, procedure skills, and medical knowledge, when devising and organizing the survey questions. We were also acutely aware of the potential impact of the pandemic on personal attitudes and a portion of the survey was dedicated to inquiring about resident well-being and burnout.

Demographic and program information was collected and included the following: clinical postgraduate year (PGY), age, race/ethnicity, gender, and residency program location (based on The American College of Obstetricians and Gynecologist (ACOG) District). With respect to patient care and procedural skills, the survey specifically queried residents about modifications to their schedules, duty hours, operative volume (major and minor surgical procedures), clinical duties, and availability and use of personal protective equipment (PPE). Residents were also asked if these changes affected their graduation requirements and overall preparedness for their postgraduate careers. When considering the impact of COVID-19 on residents’ medical knowledge, we asked about changes to educational curricula and their impact on rotation evaluations and CREOG scores. Finally, we attempted to determine the psychological effect of the pandemic on resident well-being and asked about resources provided by residency programs to combat potential burnout. The full survey is available for review in Appendix 1.

Descriptive analysis was used to summarize the data. Statistical analysis was performed using χ^2^ test or Fisher’s exact test as appropriate for categorical data and Wilcoxon rank sum test for continuous data. *P* values of < 0.05 were considered significant. All analyses were performed using multiprocessor Stata 17.0 (StataCorp LP, College Station, Texas).

## Results

One hundred thirty-five OBGYN residents initiated the survey; however, only 95 residents completed the survey in its entirety. All respondents were vaccinated and trained at programs representing each ACOG District. The respondent demographics are detailed in Table 1. The majority of participants (*n* = 61, 64.2%) were senior OBGYN residents (i.e., PGY3 or PGY4). Eighteen (18.9%) residents self-identified as PGY1s and 16 (16.8%) as PGY2s. Most were between 25 and 34 years of age (*n* = 88, 92.6%) and more than half of the residents (*n* = 52, 54.7%) self-identified as under-represented minorities (i.e., Black or LatinX). Thirty-two (33.7%) residents had been personally infected by COVID-19, and 38 (40%) had immediate household contacts who contracted COVID-19.

**Table 1:** Survey respondent characteristics

Characteristic	Number = 95 (%)
**Clinical postgraduate year (PGY)**	
PGY1	18 (18.9)
PGY2	16 (16.8)
PGY3	34 (35.8)
PGY4	27 (28.4)
**Age**	
25–29	47 (50)
30–34	41 (43.6)
35–39	5 (5.3)
40+	1 (1.1)
**Race**	
Black or African American	47 (50.5)
Caucasian or White	41 (44.1)
Latine or LatinX	5 (5.4)
**Residency Program ACOG District**	
District 1	9 (9.6)
District II	13 (13.8)
District III	12 (12.8)
District IV	19 (20.2)
District V	16 (17.0)
District VI	4 (4.3)
District VII	2 (2.1)
District VIII	4 (4.3)
District IX	5 (5.3)
District X	5 (5.3)
District XI	5 (5.3)
District XII	5.(5.3)

Eighty-two (86.3%) residents felt that their residency training had been adversely affected by COVID-19, and 70 (73.7%) had an interruption in their regularly scheduled residency training; however, over 75% (*n* = 76) of resident participants believed that their CREOG scores and rotational evaluations were unchanged during the pandemic. With respect to their procedural training, most residents (*n* = 75, 78.9%) did not think their obstetrical training had been deleteriously affected, while over 80% (*n* = 81) of residents felt that their gynecological training had suffered. Moreover, over half (*n* = 55, 57.9%) of respondents trained at institutions where restrictions were placed on gynecological procedures for greater than eight weeks. The approximate numbers of gynecological procedures performed by residents by clinical postgraduate year are illustrated in Table 2. OBGYN minimum numbers (which represent what the ACGME Review Committee [14] believes to be an acceptable minimal experience for OBGYN residents) are listed as well for reference. Of note, self-reported obstetrical numbers by clinical postgraduate year are described in Supplemental Table 1 for additional review.

**Table 2:** Self-reported approximate gynecological procedure numbers

Gynecological Procedure	PGY1	PGY2	PGY3	PGY4	p value	Minimums^*^
*Abdominal hysterectomy*	0	1 (0, 2)	8 (5, 14)	19.5 (15.25, 25)	< 0.001	15
*Vaginal hysterectomy*	0	0.5 (0, 1)	4 (2, 10)	15 (10.75, 16.75)	< 0.001	15
*Laparoscopic hysterectomy*	0 (0, 1.75)	0.5 (0, 2)	23.5 (12, 30)	53.5 (40, 69.75)	< 0.001	15
*Laparoscopy*	10 (4.25, 15)	40 (20, 52.75)	50 (40, 70)	100 (70, 100)	< 0.001	60
*Surgery for invasive cancer*	0 (0, 2)	0.5 (0, 4.25)	15 (10, 25)	26.5 (25, 40)	< 0.001	25
*Hysteroscopy*	20 (6, 28)	37.5 (30, 60)	50 (40, 57.75)	70 (50, 94.5)	< 0.001	40
*Incontinence and pelvic floor procedure*	0 (0, 1.5)	2 (0, 3.25)	15 (3.5, 24.5)	25.5 (20.5, 30)	< 0.001	25

Data are Median (IQR)

^*^OBGYN minimum numbers: OBGYN minimum numbers represent what the ACGME Review Committee [14] believes to be an acceptable minimal experience

As expected, there were significant differences in approximate gynecological numbers by clinical postgraduate year, with increasing numbers from PGY1 to PGY4 (*p* < 0.001). Notably, the median procedure numbers among 4th-year residents were all above the minimum ACGME requirements; however, the lower quartile of self-reported gynecological numbers for vaginal hysterectomies (15 (10.75–16.75)) and incontinence and pelvic floor procedures (25.5 (20.5–30)) were below the minimum ACGME requirements, indicating that the lower quartile of PGY4 respondents were likely not meeting these gynecological procedure minimums.

When asked about reaching their ACGME minimums, over a third (*n* = 40, 42.1%) of respondents were unsure if they would be able to achieve these minimum requirements by graduation. Moreover, almost 65% (*n* = 60) of residents stated that they were not confident they could practice gynecology independently upon graduation. In contrast, approximately 87% (*n* = 83) of OBGYN respondents believed they were poised to practice obstetrics autonomously following residency.

Responses from 4th-year residents to these survey questions are specifically examined in Table 3. When analyzing the responses of those PGY4 respondents who were worried they would not reach their ACGME minimums by graduation, a significant proportion of residents were not confident in their ability to practice gynecology independently following graduation. Namely, of the 27 4th-year OBGYN respondents, seven (26%) were not certain they would attain their ACGME minimums, and of those seven, over 70% (*n* = 5) did not feel prepared for autonomous practice of gynecology following graduation. Of those PGY4 residents who thought they would attain their ACGME minimums (*n* = 20, 74%), approximately 80% (*n* = 4) felt assured about their self-directed performance of gynecologic procedures after residency. These findings illustrate that there is a significant difference in the proportion of 4th-year residents ready for independent practice in gynecology depending on their ability to meet their ACGME requirements by graduation from residency (*p* = 0.013). This difference did not persist when investigating respondents’ confidence in independent post-graduation obstetrics practice and meeting ACGME minimum requirements (*p* = 0.756).

**Table 3:** Concern about reaching ACGME minimums and readiness for independent gynecological practice

Question	ACGME minimums^***^		
	Yes *n* = 20	No *n* = 7	*p* value
**Ready for independent practice in OB** ^*^			
Agree	18 (90)	6 (85.7)	0.756
Disagree	2 (10)	1 (14.3)	
**Ready for independent practice in Gyn** ^**^			
Agree	16 (80)	2 (28.6)	0.013
Disagree	4 (20)	5 (71.4)	

Data are n (%)

^*^Upon graduation, I will be ready for independent practice in general obstetrics

^**^Upon graduation, I will be ready for independent practice in general gynecology

^***^By the end of your chief year will you reach your ACGME minimums?

A significant portion of the survey attempted to determine the psychological effect of the pandemic on resident well-being. When asked about the use of personal protective equipment (PPE) when caring for patients infected with COVID-19, 20% (*n* = 15) of residents reported they did not have access to adequate PPE. Forty-five (47.3%) respondents reported violating the 80-hour per week duty requirement, and 15 residents (15.8%) reported having less than four days off per month on average during the pandemic. Over 45% (*n* = 44) of OBGYN residents conveyed that the pandemic interfered with their ability to perform at work, and 80% (*n* = 76) stated that COVID-19 had adversely influenced their mental health. Notably, 31 (32.6%) participants maintained that they had, or knew another OBGYN resident that had suicidal thoughts or had attempted self-harm or suicide.

Additional questions inquired about the support provided by residency programs and institutions to combat burnout. Over 70% of residents (*n* = 67) considered their residency leadership supportive of their wellness and education during the pandemic. Moreover, 80 (84.2%) OBGYN respondents stated that their institution had mentalhealth resources available; however, only 28 (29.4%) of residents utilized such resources.

The data on residents’ perceptions of the pandemic’s impact on mental health and their ability to perform at work (a measure used to indicate burnout) is correlated with their views on residency support, suicidal thoughts, access to wellness resources, and utilization of mental health services, as presented in Table 4. Of those residents (*n* = 76) who communicated the negative influence of the epidemic on their mental health, approximately 40% (*n* = 31) had thoughts of or knew a fellow OBGYN resident who had had thoughts of self-harm, or even potentially attempted suicide (*p* < 0.001). This significant finding persisted among those residents who suffered from burnout (*n* = 44) as almost half (*n* = 19, 43.2%) of those residents reported suicidal thoughts or actions either themselves or among those within their residency program (*p* = 0.048). In contrast, of those residents who affirmed that their mental health was unaffected by the pandemic (*n* = 18), none communicated suicidal thoughts or attempted self-harm.

**Table 4:** COVID-19 impact on OBGYN resident mental health and burnout

Question	COVID-19 impact on mental health^++^			COVID-19 impact on burnout^^^^		
	Agree *n* = 76	Disagree *n* = 18	*p* value	Agree *n* = 44	Disagree *n* = 50	*p* value
**Res leadership support of education** ^*^						
Yes	51 (67.1)	15 (83.3)	0.175	27 (61.4)	39 (78.0)	0.078
No	25 (32.9)	3 (16.7)		17 (38.6)	11 (22.0)	
**Res leadership support mental health** ^**^						
Yes	53 (69.7)	15 (83.3)	0.246	27 (61.4)	41 (82.0)	0.026
No	23 (30.3)	3 (16.7)		17 (38.6)	9 (18.0)	
**Institution resources for mental health** ^***^						
Yes	63 (84.0)	17 (94.4)	0.251	36 (83.7)	44 (88.0)	0.553
No	12 (16.0)	1 (5.6)		7 (16.3)	6 (12.0)	
**Utilization of mental health resources** ^**^**^						
Agree	23 (30.3)	4 (22.2)	0.498	15 (34.1)	12 (24.0)	0.281
Disagree	53 (69.7)	14 (77.8)		29 (65.9)	38 (76.0)	
**Suicidal thoughts or self-harm** ^**+**^						
Yes	31 (40.8)	0 (0)	< 0.001	19 (43.2)	12 (24.0)	0.048
No	45 (59.2)	18 (100)		25 (56.8)	38 (76.0)	

Data are n (%)

^*^My residency program directors/leadership have been supportive during the COVID-19 pandemic by prioritizing opportunities for education

^**^My residency program directors/leadership have been supportive during the COVID-19 pandemic by supporting wellness efforts/mental health education

^***^Does your institution have resources available for trainees dealing with mental health struggles?

^^^I have utilized the resources of my program and/or institution for dealing with my mental health during the COVID-19 pandemic.

^+^Have you, or any OBGYN resident that you know, ever had suicidal thoughts, or attempted self-harm or committed suicide during the COVID-19 pandemic?

^++^The COVID-19 pandemic has adversely impacted my mental health

^^^^The COVID-19 pandemic has interfered with my ability to perform at work

## Discussion

Our data demonstrates that COVID-19 has had a grave academic and psychologic impact on OBGYN residents across the country. Procedural training in gynecology was particularly impacted. Over 80% residents reported that their gynecological training had suffered and over half of respondents trained at institutions where restrictions were placed on gynecological procedures for greater than eight weeks. When asked about attaining their ACGME minimums, over a third of residents were unsure if they would be able to achieve these requirements in gynecology by graduation, and approximately two-thirds of respondents stated that they were not confident that they would be able practice gynecology independently following graduation from residency. When concentrating on graduating (i.e., 4th-year) resident responses, there was a significant difference in the proportion of residents reporting readiness for intendent practice in gynecology depending on their ability to meet their ACGME requirements by graduation from residency.

Resident mentalhealth was also negatively altered by the pandemic. Nearly half of OBGYN residents reported that the pandemic interfered with their ability to perform at work. While over two-thirds of residents stated that their institution had mentalhealth resources available, less than a third of residents utilized such resources. Most notably, almost a third of residents maintained that they had, or knew another OBGYN resident that had, suicidal thoughts or had attempted self-harm or suicide– emphasizing the profound psychological effect of the pandemic.

Our pilot study contributes to the emerging body of research on the effects of the COVID-19 pandemic on OBGYN residents [8–10]. It corroborates findings from Europe, where OBGYN residents experienced reduced surgical training and teaching, leading to concerns about the quality of patient care [15]. Work by Harzif et al., also complements this by examining the psychological impact (i.e., anxiety, depression, and psychological trauma) of the pandemic on Indonesian OBGYN residents [16]. Additionally, a cross-sectional survey by Winkle et al., delved into if residents’ self-reported experiences of burnout and other issues, such as depression, binge drinking, and drug use, vary according to their personal activities, including hobbies [17]. Further research suggested that resident-led wellness initiatives, like providing discretionary time and promoting social events, were the highest rated in supporting resident wellness [18]. Akin to our study findings, Wadell et al., found residents worried about the pandemic’s detrimental effects on their training, particularly among senior residents [19]. This anxiety is intensified by a national decrease in gynecologic surgeries and fellowship directors’ reports of new fellows’ unpreparedness for independent surgical practice [20, 21].

Our pilot study has several strengths and is the first of its kind to examine the impact of COVID-19 on OBGYN trainees in the United States. Our survey was conducted nationally with representation from respondents training at centers in each of the ACOG districts at a time when the direct effects of the pandemic on training were either ongoing or still very fresh in respondents’ memories, minimizing the impact of recall bias. Furthermore, while small, more than half of the residents self-identified as underfrepresented minorities, indicative of a diverse respondent population. The findings in our pilot study are suggestive of associations that should be replicated in larger samples.

Nonetheless, our pilot study has limitations, namely our low overall response rate and potential for selection bias. It is unclear how many residents received access to the survey, as there was limited verification from residency program leadership confirming distribution. Significant differences between responders and non-responders could have been overlooked. Our use of a volunteer population may not be representative of the general OBGYN resident population, and it is possible that those residents who felt more strongly about their experiences were more likely to respond. Our pilot study was also not longitudinal and cannot be translated to assess long-term effects.

## Conclusion

The COVID-19 pandemic has left virtually no one unharmed. Resident trainees, in particular, have been forced to reexamine their daily lives and practice. OBGYN residents in the United States reported concerns about their abilities for autonomous gynecological practice upon completion of residency, lending us the opportunity to provide increased support to new graduates through both formal and informal mentorship. Other potential solutions include both institutional and national working groups on gynecological procedural minimums and considerations of more flexible curriculums such as tracking. Efforts could also be made to develop surgical simulation training programs so trainees can maximize their surgical learning in the operating room. Respondents also conveyed that the pandemic deleteriously affected their mentalhealth, and while support was provided by their residency programs with resources available at their institutions to combat burnout, few residents utilized such resources. A promising solution includes the broader adoption of institution-based wellness programs and increased flexibility and time-off within clinical training to make use of institutional resources. Further large-scale investigations verifying these findings are critical.

### Electronic supplementary material

Below is the link to the electronic supplementary material.

Supplementary Material 1

Supplementary Material 2

## Data Availability

Data and materials can be obtained from the corresponding author upon reasonable request.

## References

[1] Guidance for maternal medicine services in the coronavirus (COVID-19). pandemic. https://www.rcog.org.uk/globalassets/documents/guidelines/2020-07-10-guidance-for-maternal-medicine.pdf. Published July 10, 2020. Accessed September 21, 2021.

[2] WHO Director-General’s. opening remarks at the media briefing on COVID-19–11 March 2020.Who.int.https://www.who.int/director-general/speech/detail/who-director-general-s-opening-remarks-at-the-media-briefing-on-covid-19----11-march-2020. Published 2021. Accessed January 17, 2022.

[3] Who coronavirus (COVID-19) dashboard. World Health Organization. https://covid19.who.int/. Accessed January 17, 2022.

[4] Accreditation Council for Graduate Medical Education. ACGME Response to Coronavirus (COVID19). Last Updated, March 18, 2020. https://acgme.org/Newsroom/NewsroomDetails/ArticleID/10111/ACGME-Response-to-the-Coronavirus-COVID-19. Accessed March 20, 2020.

[5] Zingaretti N, Negrini C, Tel F (2020). The impact of COVID-19 on plastic surgery residency training. Aesth Plast Surg.

[6] Huntley RE, Ludwig DC, Dillon JK (2020). Early effects of covid-19 on oral and maxillofacial surgery residency training—results from a national survey. J Oral Maxillofac Surg.

[7] Aziz H, James T, Remulla D (2021). Effect of covid-19 on surgical training across the United States: a national survey of general surgery residents. J Surg Educ.

[8] Bitonti G, Palumbo AR, Gallo C (2020). Being an obstetrics and gynaecology resident during the covid-19: impact of the pandemic on the residency training program. Eur J Obstet Gynecol Reproductive Biology.

[9] Yalçın Bahat P, Aldıkaçtıoğlu Talmaç M, Bestel A, Topbas Selcuki NF, Karadeniz O, Polat I (2020). Evaluating the effects of the COVID-19 pandemic on the physical and mental well-being of obstetricians and gynecologists in Turkey. Int J Gynaecol Obstet.

[10] Gothwal M, Singh P, Sharma C, Yadav G, Gupta MK (2022). Impact of COVID-19 pandemic on obstetrics and gynecology residency training program in India: a national online survey. J Obstet Gynaecol Res.

[11] Residency APGO. (2021, April 8). Retrieved December 29, 2022, from https://tools.apgo.org/residency-directory/.

[12] Table B3: Number of active residents, by type of medical school, GME specialty, and sex. AAMC. https://www.aamc.org/data-reports/students-residents/data/table-b3-number-active-residents-type-medical-school-gme-specialty-and-sex. Accessed March 26, 2023.

[13] Milestones guidebook for residents and fellows - ACGME. https://www.acgme.org/globalassets/PDFs/Milestones/MilestonesGuidebookforResidentsFellows.pdf. Accessed November 28, 2022.

[14] Case log information: Obstetrics and gynecology - ACGME. https://www.acgme.org/globalassets/pfassets/programresources/obgyncaseloginfo.pdf. Accessed December 12, 2022.

[15] Boekhorst F, Khattak H, Topcu EG, Horala A, Goncalves Henriques M. The influence of the COVID-19 outbreak on European trainees in obstetrics and gynaecology: A survey of the impact on training and trainee. Eur J Obstet Gynecol Reprod Biol. 2021;261:52–58. 10.1016/j.ejogrb.2021.04.005. 10.1016/j.ejogrb.2021.04.005 PMC8035806 33892209

[16] Harzif AK, Lukman DDS, Maidarti M, et al. Social factors influence on anxiety, depression level and psychological trauma of obstetrics and gynecology residents during COVID-19 pandemic. Heliyon. 2022;8(12):e12271. 10.1016/j.heliyon.2022.e12271. 10.1016/j.heliyon.2022.e12271 PMC9749376 36531625

[17] Winkel AF, Woodland MB, Nguyen AT, Morgan HK. Associations Between Residents’ Personal Behaviors and Wellness: A National Survey of Obstetrics and Gynecology Residents. J Surg Educ. 2020;77(1):40–44. 10.1016/j.jsurg.2019.08.014. 10.1016/j.jsurg.2019.08.014 31492641

[18] Seeland GR, Williams BM, Yadav M, et al. Implementation and Evaluation of a Comprehensive Resident Wellness Curriculum During the COVID-19 Pandemic. J Surg Educ. Published online December 21, 2023. 10.1016/j.jsurg.2023.11.014. 10.1016/j.jsurg.2023.11.014 38135549

[19] Wadell M, Ortqvist AK, Linden K, et al. Challenges imposed by the COVID-19 pandemic on the Obstetrics and Gynecology residency program: a mixed-methods Swedish survey in the COPE Staff cohort study. BMC Med Educ. 2022;22(1):602. Published 2022 Aug 5. 10.1186/s12909-022-03631-0. 10.1186/s12909-022-03631-0 PMC9354310 35927725

[20] Wright JD, Herzog TJ, Tsui J, et al. Nationwide trends in the performance of inpatient hysterectomy in the United States. Obstet Gynecol. 2013;122(2 Pt 1):233–241. 10.1097/AOG.0b013e318299a6cf. 10.1097/AOG.0b013e318299a6cf PMC3913114 23969789

[21] Guntupalli SR, Doo DW, Guy M, et al. Preparedness of Obstetrics and Gynecology Residents for Fellowship Training. Obstet Gynecol. 2015;126(3):559–568. 10.1097/AOG.0000000000000999. 10.1097/AOG.0000000000000999 26244537

