# Supplementary material for: The COVID-19 pandemic and OBGYN residency training: We have a problem and it’s not just masks
Source: BMC Med Educ. 2024 Apr 5;24:377. doi: 10.1186/s12909-024-05364-8 (PMC10998311; doi:10.1186/s12909-024-05364-8)
Supplement: Supplementary file 1 — Supplementary Material 1 [file 12909_2024_5364_MOESM1_ESM.docx]

Supplementary Table 1: Self-reported approximate obstetrical procedure numbers

| **Obstetric Procedures** | **PGY1^*^** | **PGY2^*^** | **PGY3^*^** | **PGY4^*^** | ***p*** | **Minimums^*^** |
| --- | --- | --- | --- | --- | --- | --- |
| *FAVD* | 0 | 0 | 2 (0, 4) | 2 (0.25, 6) | < .001 | ** |
| *VAVD* | 1 (0, 2) | 1.5 (0, 4) | 7.5 (5, 13.5) | 13 (0.25, 15.00) | < .001 | ** |
| *SVD* | 100 (70, 110) | 182.5 (147.5, 218.75) | 250 (200, 300) | 270 (250, 335) | < .001 | 200 |
| *Cesarean delivery* | 27.5 (9.25, 41.25) | 100 (58.25, 133.75) | 165 (122.5, 221) | 250 (200, 259.50) | < .001 | 145 |
| *Abortion* | 7.5 (4.25, 15) | 19 (10, 40) | 50 (30, 53) | 50 (30, 69.5) | < .001 | 20 |

FAVD: forceps assisted vaginal delivery, VAVD: vacuum assisted vaginal delivery, SVD: spontaneous vaginal delivery

Data are Median (IQR)

^*^OBGYN minimum numbers: OBGYN minimum numbers represent what the ACGME Review Committee^12^ believes to be an acceptable minimal experience. These are listed for reference.

^**^15 operative vaginal deliveries total (including both FAVD and VAVD)
