# Supplementary material for: The COVID-19 pandemic and OBGYN residency training: We have a problem and it’s not just masks
Source: BMC Med Educ. 2024 Apr 5;24:377. doi: 10.1186/s12909-024-05364-8 (PMC10998311; doi:10.1186/s12909-024-05364-8)
Supplement: Supplementary file 2 — Supplementary Material 2 [file 12909_2024_5364_MOESM2_ESM.docx]

**Appendix 1:**

1. My residency training has been adversely impacted by the COVID-19 pandemic.
2. Strongly agree
3. Agree
4. Neutral
5. Disagree
6. Strongly disagree
7. I had interruption of my regularly scheduled residency training due to the COVID-19 pandemic.
   1. Yes
   2. No
   3. Not sure
8. My obstetrical training has been adversely impacted by the COVID-19 pandemic.
   1. Strongly agree
   2. Agree
   3. Neutral
   4. Disagree
   5. Strongly disagree
9. Number of obstetrical procedures (Estimate:
   1. FAVD
   2. VAVD
   3. Vaginal delivery
   4. Cesarean delivery
   5. Cesarean hysterectomy
10. My gynecologic training has been adversely impacted by the COVID-19 pandemic.
    1. Strongly agree
    2. Agree
    3. Neutral
    4. Disagree
    5. Strongly disagree
11. Numbers of gynecologic procedures (Estimate):
    1. TAH
    2. TVH
    3. Laparoscopic hysterectomy
    4. Diagnostic and Operative L/S
    5. Surgery for invasive cancer
    6. Diagnostic and Operative hysterectomy
    7. Robotic surgery
    8. Incontinence and pelvic floor procedure
12. Have gynecological procedures been restricted at your institution?
    1. Yes
    2. No
    3. Not sure
13. If restrictions have been placed on gynecological procedures at your institution, how long have they been in place?
    1. Approximately 2 weeks
    2. Approximately 4 weeks
    3. Approximately 6 weeks
    4. Approximately 8 weeks
    5. Greater than 8 weeks
14. Have your CREOG scores been impacted by the COVID-19 pandemic?
    1. Yes
    2. No
    3. Not sure
15. Have your rotational evaluations been impacted by the COVID-19 pandemic?
    1. Yes
    2. No
    3. Not sure
16. By the end of your chief year, will you reach your ACGME minimums?
    1. Yes
    2. No
    3. Not sure
17. Upon graduation, I am ready for independent practice in general obstetrics.
    1. Strongly agree
    2. Agree
    3. Neutral
    4. Disagree
    5. Strongly disagree
18. Upon graduation, I am ready for independent practice in general gynecology.
    1. Strongly agree
    2. Agree
    3. Neutral
    4. Disagree
    5. Strongly disagree
19. How many days off per month on average do you have during the COVID-19 pandemic?
    1. Less than 4 days off per month
    2. 4 days off per month
    3. Greater than 4 days off per month
20. Have you violated the 80-hour per week duty requirement during the COVID-19 pandemic?
    1. Yes
    2. No
    3. Not sure
21. When caring for patients with COVID-19, were you provided adequate PPE?
    1. Yes
    2. No
    3. Not sure
22. The COVID-19 pandemic has adversely impacted my mental health and has interfered with my ability to perform at work.
    1. Strongly agree
    2. Agree
    3. Neutral
    4. Disagree
    5. Strongly disagree
23. My residency program directors/leadership have been supportive during the COVID-19 pandemic by prioritizing opportunities for education.
    1. Yes
    2. No
    3. Not sure
24. My residency program directors/leadership have been supportive during the COVID-19 pandemic by supporting wellness efforts/mental health education.
    1. Yes
    2. No
    3. Not sure
25. Does your institution have resources available for trainees dealing with mental health struggles?
    1. Yes
    2. No
    3. Not sure
26. I have utilized the resources of my program and/or institution for dealing with my mental health during the COVID-19 pandemic
    1. Strongly agree
    2. Agree
    3. Neutral
    4. Disagree
    5. Strongly disagree
27. Have you, or any OBGYN resident that you know, ever had suicidal thoughts, or attempted self-harm or committed suicide during the COVID-19 pandemic?
    1. Yes
    2. No
    3. Not sure
28. If I could do it all over again, I would still pursue a career in OBGYN.
    1. Strongly agree
    2. Agree
    3. Neutral
    4. Disagree
    5. Strongly disagree
29. If I could do it all over again, I would still pursue a career in MEDICINE itself.
    1. Strongly agree
    2. Agree
    3. Neutral
    4. Disagree
    5. Strongly disagree
30. Have you been infected with COVID-19 yourself?
    1. Yes
    2. No
    3. Not sure
31. Have your immediate household contacts been infected with COVID-19?
    1. Yes
    2. No
    3. Not sure
32. Are you vaccinated?
    1. Yes
    2. No
    3. Not sure
33. How else has the COVID-19 pandemic impacted you as a resident? (free response)

**DEMOGRAPHICS:**

1. Residency year
   1. PGY1
   2. PGY2
   3. PGY3
   4. PGY4
2. Gender
   1. Cis Male
   2. Cis Female
   3. Trans male
   4. Trans female
   5. Non-binary
   6. Gender non-conforming
   7. Prefer not to answer
3. Age
   1. 21-24
   2. 25-29
   3. 30-34
   4. 35-39
   5. 40-44
   6. 45-49
   7. 50+
4. Race
   1. Asian or Pacific Islander
   2. Black or African-American
   3. Caucasian or White
   4. Latine or Latinx
   5. Native American or Alaskan Native
   6. Biracial
5. Where is your residency program located?
   1. ACOG District I (Atlantic Provinces, Chile, Connecticut, Maine, Massachusetts, Quebec, Rhode Island, and Vermont)
   2. ACOG District II (New York and Bermuda)
   3. ACOG District III (Delaware, Dominican Republic, New Jersey, and Pennsylvania
   4. ACOG District IV (District of Columbia, Georgia, Maryland, North Carolina, South Carolina, Virginia, West Virginia, Argentina, Puerto Rico and the West Indies
   5. ACOG District V (Indiana, Kentucky, Ohio, Michigan, and Ontario
   6. ACOG District VI (Illinois, Iowa, Minnesota, Nebraska, North Dakota, South Dakota, Wisconsin, Manitoba, Saskatchewan, and Peru
   7. ACOG District VII (Alabama, Arkansas, Kansas, Louisiana, Mexico, Mississippi, Missouri, Oklahoma, and Tennessee).
   8. ACOG District VIII (Alaska, Alberta, Arizona, British Columbia, Central America, Colorado, Hawaii, Idaho, Montana, Nevada, New Mexico, Oregon, Utah, Washington, Wyoming, American Samoa, Guam, Northwest Territory, and Yukon Territory)
   9. ACOG District IX (California and Ecuador)
   10. Armed Forces District
   11. ACOG District XI (Texas)
   12. ACOG District XII (Florida and Colombia)
